# UHPLC Enantiomer Resolution for the ɑ/β-Adrenoceptor Antagonist *R/S*-Carvedilol and Its Major Active Metabolites on Chiralpak IB N-5

**DOI:** 10.3390/molecules27154998

**Published:** 2022-08-05

**Authors:** Liza Samir, Rasha Hanafi, Sami El Deeb, Hilde Spahn-Langguth

**Affiliations:** 1 Pharmaceutical Chemistry Department, Faculty of Pharmacy and Biotechnology, German University in Cairo, Cairo 11865, Egypt; 2 Institute of Medicinal and Pharmaceutical Chemistry, Technische Universität Braunschweig, 38106 Braunschweig, Germany; 3 Natural and Medical Sciences Research Center, University of Nizwa, P.O. Box 33, Birkat Al Mauz, Nizwa 616, Sultanate of Oman; 4 Institute of Pharmacy and Biochemistry-Therapeutic Life Sciences, Johannes Gutenberg-University Mainz, 55128 Mainz, Germany

**Keywords:** quality-by-design, chiral separation, carvedilol, active metabolites, docking, cardiovascular patients

## Abstract

Carvedilol (CAR), a racemic lipophilic aryloxy propanolamine, acts as a selective α_1_-adrenoreceptor antagonist and a nonselective β-adrenoreceptor antagonist. CAR metabolism mainly produces three active metabolites: desmethyl carvedilol (DMC), 4′-hydroxy carvedilol (4′OHC) and 5′-hydroxy carvedilol (5′OHC). The oxidative *S*-(-)-metabolites contribute to the β-antagonistic effect, yet not to the α-antagonistic effect to be observed after drug dosage. Therefore, the three β-adrenoceptor blocking metabolites, which are structurally closely related to the parent CAR, are included into the development of a bioanalytical quantitative method for all major active species relevant with respect to adrenoceptor-blockade. Because of the given pharmacological profile, resolution of the enantiomers of carvedilol, of 4′- and 5′-hydroxy carvedilol as well as of DMC, is mandatory. The current study aims to determine the response surface for the enantiomer separation of the parent CAR as well as the major metabolites on a suitable chiral stationary phase. Design of experiment approach (DoE) was utilized in an initial screening phase followed by central-composite design for delimitation of the response surface for resolution of the four enantiomeric pairs in least run time. The impact of chromatographic variables (composition and percentage of organic modifier(s), buffer type, buffer pH, flow rate) on critical peaks resolution and adjusted retention time was evaluated, in order to select the most significant critical quality attributes. On this basis, a robust UHPLC-UV method was developed and optimized for the simultaneous, enantioselective determination of CAR along with its major active metabolites (4′OHC, 5′OHC, and DMC) on Chiralpak IBN-5. The optimized UHPLC-UV method (which includes metoprolol as the internal standard) was validated according to the ICH M10 guidelines for bioanalytical methods and proven to be linear, precise, accurate, and robust. The validated assay was applied to plasma samples from cardiovascular patients treated with *rac*-CAR (blood randomly drawn at different times after oral CAR intake). In order to provide more insight into the mechanism of the enantiomer separation of CAR and its metabolites on the CSP, docking experiments were performed. Molecular simulation studies suggest the chiral recognition to be mainly due to different binding poses of enantiomers of the same compound.

## 1. Introduction

**Figure 1:**
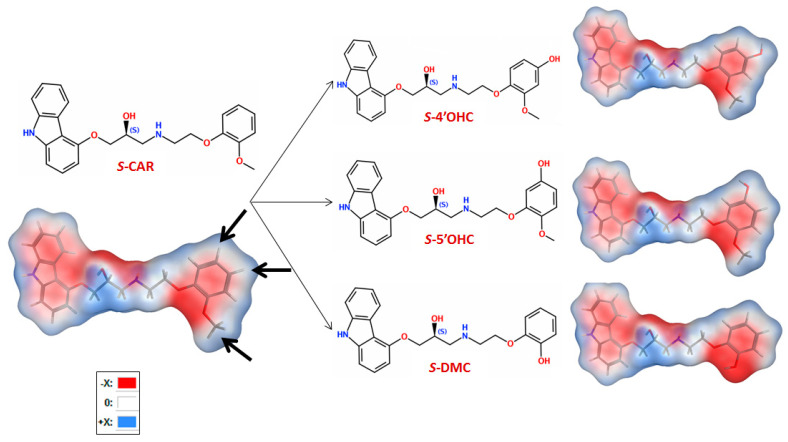
2D structures of *S*-CAR ((-)-1-(9*H*-carbazol-4-yloxy)-3-[2-(2-methoxyphenoxy) ethylamino] propan-2-ol) (**left**) with its more hydrophilic and active metabolites (*S*-*DMC*, *S*-4′OHC and *S*-5′OHC) (**right**) and energy-minimized 3D structure (TPSA, with electrostatic map) of *S*-CAR (**left**) and 3D images of *S*-DMC, *S*-4′OHC and *S*-5′OHC (**right**).

Carvedilol (CAR), a racemic lipophilic aryloxy propanolamine, has become the first approved adrenoceptor antagonist for the treatment of congestive heart failure on the basis of several clinical trials (e.g., COMET) [1]. CAR was initially designed as a ‘hybrid drug’ combining α-and β-adrenoceptor-antagonistic properties. It has a relatively high molar mass of 406.5 g/mol. CAR is a BCS class II drug, has one chiral center within its molecule and is commercially available as a racemate (Figure 1). It acts as a selective α_1_-adrenoreceptor antagonist with an apparent dissociation constant (*K_d_*) of 0.9 nmol/L and a nonselective β-adrenoreceptor antagonist with a *K_d_* of 11 nmol/L [2]. The ratio of α_1_- and β-adrenoceptor blockade of CAR is 1:10.

Carvedilol’s β-adrenoreceptor antagonistic property resides in the *S*-(-)-enantiomer with a *K_d_* of 0.4 nmol/L making it approximately two-fold more potent than the racemic mixture [3]. On the other hand, the *R-*(+)-enantiomer has only approximately 1% of the β-blocking activity associated with the racemate (*K_d_* about 45 nmol/L). With respect to the α_1_-antagonistic activity, both, *R*- and *S*-enantiomers exhibit almost identical activity (*K_d_* 16 and 14 nmol/L, respectively) [3,4,5]. Understanding this complex profile with activation as well as deactivation upon metabolism requires knowledge of the respective eudismic ratios: The β-adrenoceptor-antagonism eudismic ratio of the parent CAR amounts to approximately 100, while its α_1_-adrenoceptor-antagonism eudismic ratio is close to 1.

The high-clearance drug CAR undergoes extensive first-pass liver metabolism with preference for the *S*-enantiomer in young healthy volunteers. Upon administration of a therapeutic dose, almost 98% of *rac*-CAR is metabolized and only 2% excreted unchanged into urine [6].

Both, phase-I and -II processes are involved in metabolic CAR clearance, and it is primarily metabolized via aromatic ring oxidation with sequential glucuronide formation. The parent drug, but also the oxidized metabolites, are subject to phase-II metabolism (via glucuronidation and sulfation [7]). Mainly three oxidative metabolites are relevant with respect to β-adrenoceptor blockade: desmethyl carvedilol (DMC), 4′-hydroxy- (4′OHC), and 5′-hydroxy carvedilol (5′OHC) (Figure 1) [7].

These major oxidative metabolites were found to retain their pharmacological activity only with respect to β_1_-adrenoceptor blockade, and as for the drug itself, also for the metabolites the eudismic ratio *S/R* was significantly higher than 1 in β_1_-specific radioreceptor assays, a pharmacotherapeutic fact supporting the necessity for an enantiospecific assay also for these metabolites [8,9,10,11,12]. However, this also means that (as opposed to the the α_1_-adrenoceptor antagonistic parent *R*-CAR) the three studied oxidative metabolites of *R*-CAR are inactive. In summary, *R*-enantiomers of CAR-metabolites are not β-adrenoceptor antagonists, and neither *S*- nor *R*-enantiomers of CAR-metabolites are vasodilating α-adrenoceptor antagonists.

Hence, CAR-species contributing to the effect in vivo are for β-adrenoceptor antagonism *S*-CAR and its metabolites and for α-adrenoceptor antagonism *S*-CAR and *R*-CAR only (CAR metabolites are no vasodilators, *R*-CAR metabolites are entirely inactive). This means that, considering the in-vivo effect profile, the two enantiomers of the parent CAR as well as the *S*-enantiomers of the three metabolites are of interest for bioanalytical procedures.

In previous studies, separation of *S*- from *R*-CAR was reported either following derivatization ([13,14,15,16,17]) or using chiral stationary phases such as cyclodextrin [18], teicoplanin [19], Chiralpak AD^TM^ [20]. E.g., Chirobiotic^TM^ V was recently used to separate each enantiomer pair, which means *R*- from *S*-CAR, *R*- from *S*-4′OHC, *R*- from *S*-5′OHC as well as *R*- from *S*-DMC upon injection as single pure *rac*-reference compounds. Separation of CAR from its metabolites was easily accomplished with various CSPs, yet the retention behavior of the three metabolites was too similar on these respective CSPs, this means that the six metabolite analytes (=enantiomeric pairs of each of the three metabolites) were not or not completely resolved, when all the *rac*-compounds were injected simultaneously. Here, the retention times of the analytes were too close, almost identical in some cases. Furthermore, in the reported Chirobiotic V-based assay, enantiomers of hydroxylated CAR metabolites were not resolved when injected simultaneously, and their concentrations were reported as total racemate concentrations [21].

CAR enantiomers were easily separated from their metabolites, the enantiomers of CAR showed baseline separation in all reported HPLC methods, but this was not the case for the conglomerate of the remaining six analytes (=enantiomers of the three metabolites). Such simultaneous assays for CAR and CAR metabolites were usually not optimized systematically. All previous approaches were based on the OFAT—where only one factor affecting the separation is varied at a time rather than systematic design of experiments (DoE). Moreover, to date, the simultaneous separation of CAR enantiomers along with its three major oxidative metabolites has not been achieved on chiral stationary phases (CSPs). In previously reported studies, either the different *rac*-analytes were resolved with no or insufficient enantiomer separation or the respective enantiomers were resolved, yet without separation of *rac*-analytes. As a consequence, mass-based quantification of different species in the LC-eluate (including fragmentation) may be needed and be employed, in order to differentiate between compounds with close physicochemical relation, such as some structural- and stereo-isomers. Differentiation of the enantiomers of 4′OHC and 5′OHC (which are characterized by a high structural similarity with an identical molar mass (422.5) and identical calculated logP value) is of particular concern, while the molar mass of DMC (392.5) lies below those of CAR (406.5) and the two OHCs.

The current study mainly aims at the development and optimization of an enantiospecific assay for simultaneous determination of the eight analytes (the parent drug and metabolites) to be included after administration of the adrenoceptor antagonist CAR as well as characterization of the kinetic properties of CAR metabolites in vivo. Considering the complex pharmacodynamic and pharmacokinetic characteristics [6,7,8,9,10,11,12,13,14,15,16,17,18,19,20,21,22], this work is of particular interest for studies -in patients/populations at risk- of enantioselectivity of (A)DME processes for the parent drug and metabolites (such as protein binding and distribution, metabolism and sequential metabolism, excretion/secretion into urine and bile) and PK/PD correlations. The major focus of this study is the optimization of chromatographic conditions (organic modifier composition and content, buffer type and pH and flow rate) through a systematic DoE. Moreover, we were interested to discover whether particular structural/physicochemical features play a role and could serve as predictive factors.

## 2. Results

### 2.1. Design of Experiment Approach for Enantiomer Resolution of CAR and Its Metabolites

#### 2.1.1. Screening Including Enantiomer Separation of the Most Critical Analyte (4′-OHC) and Total Run-Time (All Analytes) as the Two Optimizable Measures

**Figure 2:**
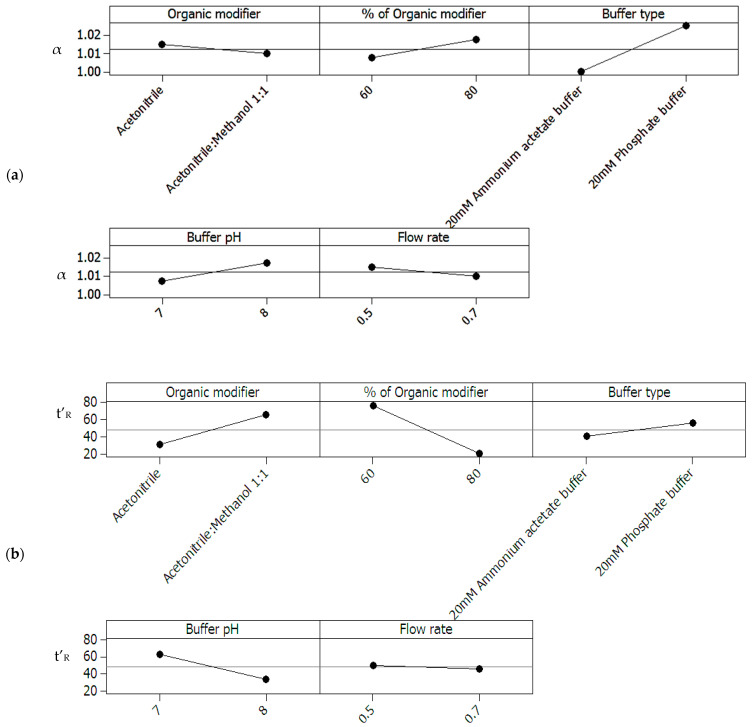
(**a**) Screening design for peak separation: main effects plots for separation factor (α) of the critical peak pair of (4′OHC) enantiomers, (**b**) Screening design for retention time: main effects plots for adjusted retention time (t’_R_) of the last eluted peak (*R*-CAR).

Separation factor (α) and adjusted retention time (t’_R_) for the eight experimental runs were used to generate main effects plots (Figure 2a,b) that show the relationship between the factors’ levels and the means of responses. Main effects plots for α (Figure 2a) showed buffer type as the factor of highest significance, followed by percentage of organic modifier and buffer pH. Organic modifier composition and flow rate are the least significant factors. Phosphate buffer showed a higher separation factor α compared to ammonium acetate at same pH values (7.0 and 8.0 for each). Organic modifier, buffer pH and buffer type were also found to affect the adjusted retention time (Figure 2b), consecutively, with acetonitrile, pH 8 and ammonium acetate being the factors with lower adjusted retention time. Flow rate had no considerable effect on the adjusted retention times. 

#### 2.1.2. General Design for the Optimization of Conditions and Respective Outcomes

Percent organic modifier (X_1_), buffer pH (X_2_) and organic modifier composition (X_3_) were further investigated using central composite design (CCD), as the most influential numerical factors according to the screening design results. Flow rate and buffer type were held constant at 0.5 mL/min and 20 mM phosphate buffer, respectively. Resolution (resolution factor, R_s_) of the critical peak pair (4′OHC enantiomers) and adjusted retention times (t’_R_) were calculated to evaluate the response for each run (Table 1).

**Figure 3:**
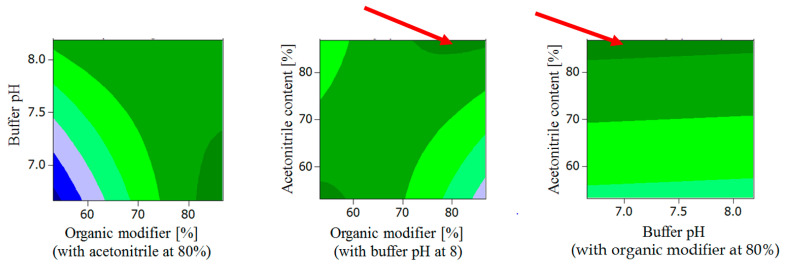
Response surface contour plots for R_s_^2^: the dark green plot regions represent those of the highest R_s_. The red arrows indicate the “global solution” predicting the optimum chromatographic conditions for highest R_s_ (see Section 2.1.5).

Response surface contour plots showed highest R_s_ for the following conditions (Figure 3):

- Buffer pH < 7.3, organic modifier > 80% with acetonitrile content in organic modifier at 80 %.
- Organic modifier < 55% with acetonitrile content in organic modifier < 55%, Buffer pH at 8.
- Organic modifier > 85% with acetonitrile content in organic modifier > 70%, Buffer pH at 8.
- Organic modifier > 85% with acetonitrile content in organic modifier at 80%, Buffer pH 6.5–8.5.

**Figure 4:**
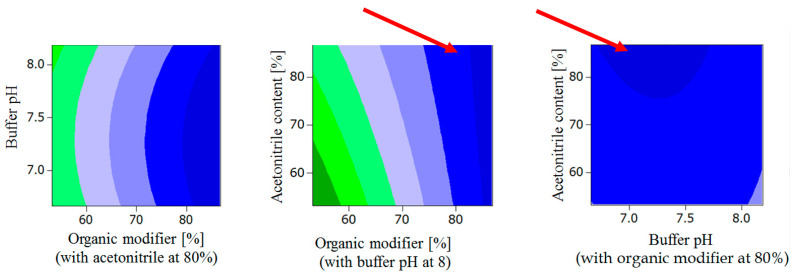
Response surface contour plots for ln t’_R_: The dark blue plot regions represent those of the highest ln t’_R_. The red arrows indicate the “global solution” predicting the optimum chromatographic conditions for lowest t’_R_ (see Section 2.1.5).

Moreover, lowest observed ln t’_R_ was observed for the following conditions (Figure 4):

- Acetonitrile content in organic modifier range 60–80%, organic modifier > 80%, Buffer pH at 8.
- Buffer pH range 6.5 to 8.2, organic modifier > 80% with acetonitrile in organic modifier at 80%.

#### 2.1.3. Regression Analysis for Further Optimization and for Elimination of Insignificant Terms

ANOVA analysis for the R_s_^2^ regression model:

A first attempt to fit the model using R_s_ showed high standard error (SE). The R_s_^2^ regression model, however, displayed SE = 0.0626, correlation coefficient (R^2^) = 88.14%, predicted correlation coefficient (R^2^_pred_) = 66.28% and adjusted correlation coefficient (R^2^_adj_) = 82.67% (Equation (1)). The R_s_^2^ regression model was the best that could be achieved, although a drop in R^2^_pred_ was observed which might be a sign of model overfitting. Attempting to increase R^2^_pred_ by reducing the number of terms caused a significant drop in R^2^.

R_s_^2^ = −11.8874 + 0.1807 X_1_ + 1.8654 X_2_ − 0.0645 X_3_ − 0.0005 X_1_ × X_1_ − 0.0235 X_1_ × X_2_ + 0.0010 X_1_ × X_3_ (1)

R_s_ = Resolution
X_1_ = Percent of organic modifier
X_2_ = Buffer pH
X_3_ = Acetonitrile percentage of organic phase

The regression model also showed a high significance at a confidence interval of 95% indicated by *p*-values with values lower than 0.05.

ANOVA analysis for t’_R_ regression model:

Since using t’_R_ as the response to fit a model showed a high s-value, a second attempt was made fitting ln t’_R_ which yielded a satisfactory model (Equation (2)). The best obtained model showed R^2^_pred_ = 97.95%, R^2^_adj_ = 98.8% and lowest SE of 0.0768. The regression model also shows high significance at a confidence interval of 95% indicated by *p*-values lower than 0.05.

ln t’_R_ = 38.6525 − 0.1489 X_1_ − 6.2581 X_2_ − 0.0916 X_3_ + 0.4278 X_2_ × X_2_ + 0.0010 X_1_ × X_3_ (2)

t’R = Adjusted retention time
X_1_ = Percent of organic modifier
X_2_ = Buffer pH
X_3_ = Acetonitrile percentage in organic modifier

**Figure 5:**
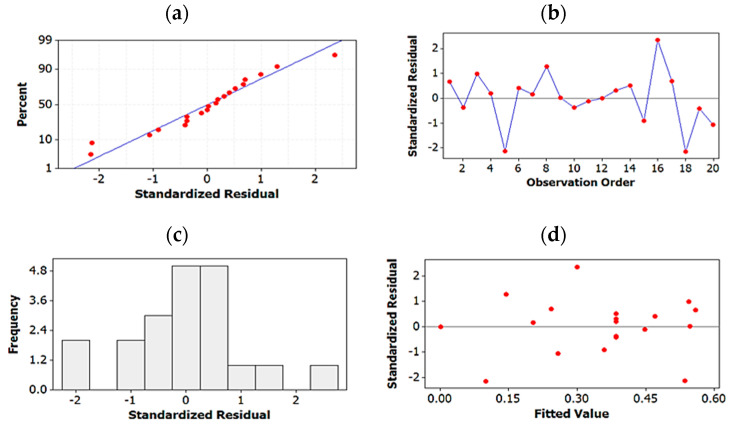
Residual plots for R_s_^2^: (**a**) normal probability plot of residuals, (**b**) residuals-vs.-fit plots, (**c**) histogram of residuals, (**d**) residuals-vs.-order of data.

**Figure 6:**
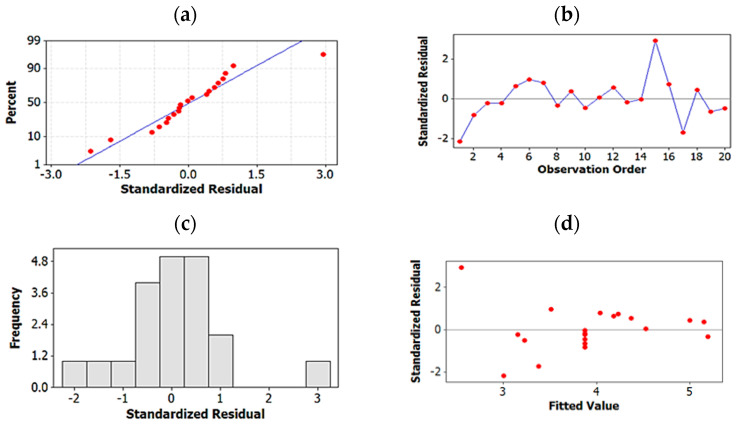
Residual plots for ln t’R: (**a**) normal probability plots of residuals, (**b**) residuals-vs.-fit plots, (**c**) histogram of residuals, (**d**) residuals-vs.-order of data.

Residual plots for the best regression models of both R_s_^2^ and ln t’_R_:

Four-in-one residual plots were generated for the regression models of both R_s_^2^ and ln t’_R_ to examine the goodness-of-fit in regression. Normal probability plots of residuals, residuals-vs.-fit plots, histogram of residuals plots and residuals-vs.-order of data were used for this purpose (Figure 5 and Figure 6). The normal probability and histogram of residuals plots show evidence of normal distribution of the residuals since the data points follow a straight line in the normal probability plots and are shown to follow normal distribution in the histograms. The residuals-vs.-fit plots indicate no evidence of non-constant variance, missing terms or the presence of outliers since all data points are randomly scattered around zero. Finally, in the residuals-vs.-order plots, points fluctuate randomly around the centerline proving that errors are independent of one another.

#### 2.1.4. Testing Model Predictability

Five experimental runs with factor levels within the robust area of highest R_s_^2^ and lowest ln t’_R_ in the contour plot (Figure 3 and Figure 4) were randomly selected and set up. The results were compared against the predicted fitted responses according to the proposed models. The percent residual values for all five runs were found to fall within the pre_s_dicted range of 95% prediction interval and the residual percent of all runs had values of within −5.9% to +6.3%.

#### 2.1.5. Global Solution: Assay Method Optimized on the Basis of Resolution and Total Run-Time

Optimum conditions that simultaneously produce the best values of resolution and shortest run time were calculated by the response optimizer tool in Minitab17 software based on the final regression models obtained. The best chromatographic conditions for enantiomeric separation of CAR and its metabolites were predicted to be as follows:

The optimized mobile phase consists of 80% organic modifier (acetonitrile 87% and methanol 13%) and 20% aqueous potassium phosphate buffer pH 7. A flow rate of 0.5 mL/min is used, and the temperature is set at 25 °C, with predicted ln t’_R_ of 2.9 (t’_R_ = 18.2 min) and R_s_^2^ of 0.659 (R_s_ = 0.8). The flow rate is set constant at 0.5 mL/min and temperature at 25 °C. 

This “global solution” for optimum chromatographic conditions falls within the robust area of the response surface predicting highest R_s_ (Figure 3) and low t’_R_ (Figure 4).

**Figure 7:**
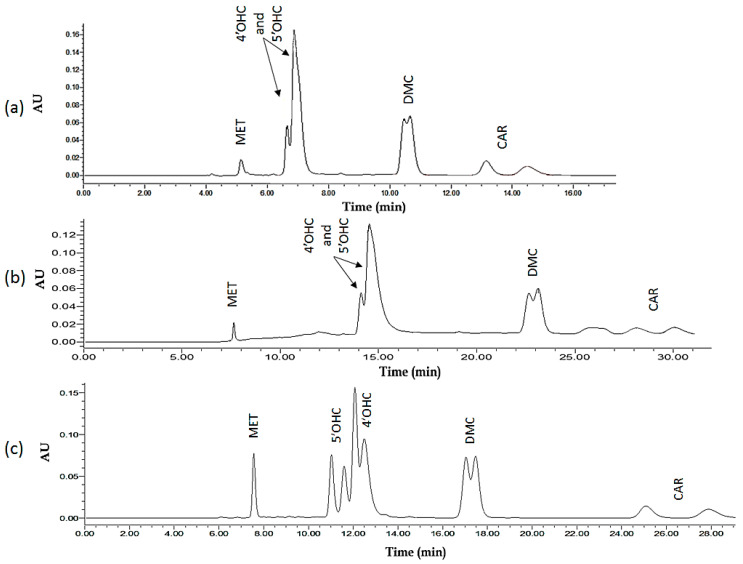
Chromatograms representing the chromatographic behaviour of the analytes and their resolution of the enantiomers of CAR and its major oxidative metabolites on Chiralpak IB N-5 as CSP using MET (*rac*-metoprolol as IS) The injection volume is 10 μL (of a 50 ng/mL solution of respective racemates): (**a**) An example for an *unoptimized* chromatogram. The mobile phase used consists of 80% organic modifier (50% acetonitrile: 50% methanol) and 20% 20 mM potassium phosphate buffer pH 8, at a flow rate of 0.7 mL/min and 25 °C. ACN (60%), Peak assignments: MET (one peak); 5′OHC and 4′OHC (two peaks); DMC (two peaks); CAR (two peaks). (**b**) A second example for an *unoptimized* chromatogram. Here, mobile phase used consists of 60% organic modifier (100% acetonitrile) and 40% 20 mM ammonium acetate buffer pH 8, at a flow rate of 0.7 mL/min and 25 °C. ACN (60%), Peak assignments: MET (one peak); 5′OHC and 4′OHC (two peaks); DMC (two peaks); CAR (two peaks). (**c**) The *‘Global solution’* mobile phase used consists of 80% organic modifier (87% acetonitrile: 13% methanol) and 20% 20 mM phosphate buffer pH 7, at a flow rate of 0.5 mL/min and 25 °C. Peak assignments: MET (one peak); 5′OHC (two peaks); 4′OHC (two peaks); DMC (two peaks); CAR (two peaks).

Figure 7 (lower part, 7c) depicts the chromatogram for a representative run using predicted optimum conditions as well as two unoptimized initial runs (Figure 7a,b). Upon routine application, the system was found to yield stable *S/R* ratios for all enantiomer pairs. These ratios approximated 1.0, showing that, in spite of resolution factors R_s_ being in part smaller than 1, the optimized assay is applicable for the enantiospecific quantification of CAR and its oxidative metabolites. This also holds true for the respective plasma assay.

### 2.2. CSP Regeneration and Validation of a Preliminary Plasma Assay

#### 2.2.1. CSP Regeneration

Although column performance was generally stable, precautions were taken to protect the column from efficiency-changing impurities and contaminations, which may accumulate with time, particularly when injecting plasma extracts. Column washing (with acetonitrile: water 50:50 *v*/*v* for 1 h at a flow rate of 1 mL/min) after 30 or 35 injections prevented the decrease in efficiency to a significant extent and permitted a long-term use of the stationary phase.

#### 2.2.2. Validation of Plasma Assay

Validation of the optimized UHPLC method was evaluated based on the requirements of the ICH guidelines [23]. The method showed good linearity for all analytes (studied as racemates with *R/S* ratio = 1) in the investigated ranges (Table 2). Regression equations and regression coefficients for the calibration curves along with LOD and LOQ values for the analytes are shown in Table 2. The mean recovery % (accuracy) of CAR, 4′OHC, 5′OHC, DMC were found to be 103.3, 99.9, 102.7, and 99.6%, respectively, proving method accuracy, with low standard error values of 1.7, 0.4, 0.3, 0.1. Because there was no difference between the enantiomers, each of the given values represents the mean of *S* and *R* for simplification, both intra- and inter-day precisions of all analytes were satisfactory with RSD < 15% for all analytes’ slope and y-intercept. The extraction recoveries (extraction yields) of CAR, 4′OHC, 5′OHC, DMC enantiomers from plasma were found to be approximately 90% for CAR enantiomers and between 76 and 78% for the enantiomers of the oxidative metabolites, respectively, (with percentage RSD values < 10 (n ≥ 3)) for the QC samples. In the investigated range, extraction yields were not found to be concentration-dependent. The respective extraction recovery of the internal standard MET amounts to 42%. The system suitability parameters were evaluated and compared to the European Pharmacopoeia Chapter 2.2.46 (system suitability requirements for Chromatographic Separation Techniques). Best R_s_ achieved for the critical peaks (4′OHC enantiomers) was found to be 0.8, which is lower than that suggested in the ICH guidelines (>1.5). R_s_ for the enantiomers of CAR, 5′OHC and DMC were: 2.4, 1.2 and 1.0, respectively. The current method achieved sharp peaks with tailing factors of (T) ≤ 1.4, which is less than the value recommended by ICH guidelines (<2). The retention factor limit (between 1 and 10) was also achieved, and the plate count was (N) > 5000. Testing the robustness of the method by visualizing the response surface obtained from the optimization design shows the area for maintaining a high resolution for percentage of organic modifier more than 85%, and buffer pH 6.5–7.3. The retention time is maintained minimum for percentage of organic modifier more than 80% and the percentage range of acetonitrile in the organic modifier (60–80%) and buffer pH 6.5–8.2 (Figure 3 and Figure 4).

### 2.3. Assay Application to Patient Plasma Samples

**Figure 8:**
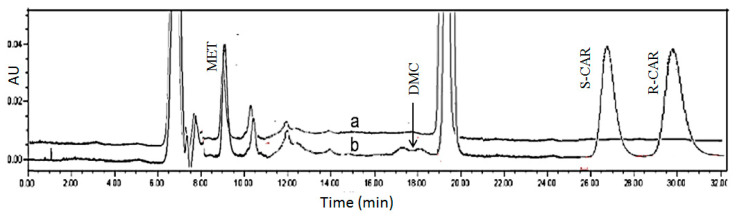
Chromatograms corresponding to (a) a blank plasma sample of patient #2 (spiked with 2.5 µL of 100 ng/mL MET (IS) stock), (b) a plasma sample of patient #2 (sample obtained 1 h following a single oral dose of *rac*-CAR (25 mg)).

The developed method was applied to single plasma samples obtained from different patients after an oral dose administration of 25 mg *rac*-CAR under non-standardized conditions. Patients’ plasma was collected at different time intervals after drug administration and CAR and its metabolites were extracted according to the method in Section 4.7 after spiking with IS. The enantiomer concentrations for parent CAR and its metabolites detected in the plasma samples of six different patients are shown in Table 3. Figure 8 shows the chromatogram corresponding to extracted plasma from a patient in comparison with standard blank plasma.

### 2.4. Computational Study: Investigation of Analyte-CSP Affinities and Identification of Possible Binding Sites and Mechanisms

**Figure 9:**
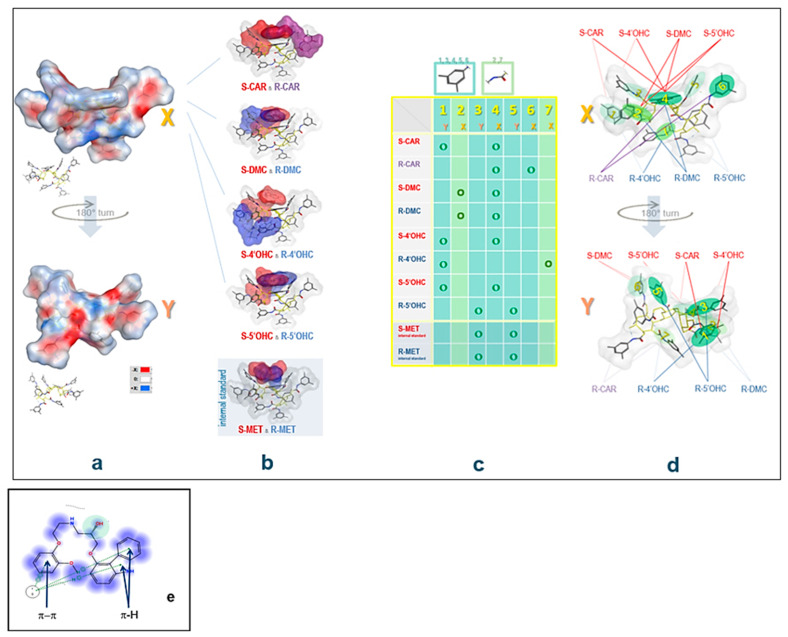
Interactions of CAR, 4′OHC, 5′OHC, and DMC (and MET) enantiomers with the CSP: Highscore docking poses and character of major binding interactions of the analytes at the CSP. Top left side (**a**,**b**): (**a**) CSP polar surface (TPSA) of the CSP 3-unit segment, front view = X, back view = Y; (**b**) analyte enantiomers of CAR, DMC, 4′OHC, and 5′OHC attached to grey CSP surface (enantiomer color code: red surface for *S*-enantiomers (all β-adrenoceptor antagonistic enantiomers, except for the α-/β-adrenoceptor antagonistic *S*-CAR), purple surface for the α_1_-adrenoceptor antagonistic *R*-enantiomer of the parent CAR, blue surface for α_1_-inactive metabolite *R*-enantiomers). Top right side (**c**,**d**): (**c**) Binding areas at the CSP: Table listing the two rim regions of high-score binding for each of the analytes, which characterize the expansion of analyte-CSP high-score docking (for all 8 analytes as well as the internal standard). There are seven major regions (1–7), where regions 1, 3, 4, 5, and 6 are representing 3,5-dimethyl phenyl residues and regions 2 and 7 the carbamate spacers. The respective location on the CSP surface is indicated, X = front, Y = back. (**d**) Location of regions 1–7 on the CSP unit: X front view, Y back view, regions 2, 4, 6, and 7 are located on the front side, regions 1, 3, and 5 on the back side. Bottom (**e**): Binding forces involved in the docking of, e.g., *S*-CAR: Binding includes H-arene and π-π stacking (for the two terminal aromatic systems) and hydrophobic interactions (for the bridging middle chain). The OH at the chiral center (marked in light green) interacts with the carbonyl in the CSP (H-bonding). For all analytes, major CSP-binding forces include π-π and H-π for the 3,5-dimethyl phenyl and dipole-dipole for the carbamate segment.

Upon docking and observing the poses produced, the enantiomers of all analytes showed a “U”-shape fold of the structure to maximize the interactions with the CSP. Different docking poses were observed with a different score of the most stable conformer for enantiomers of the same molecule (Figure 9). Ligand interactions between the best docking pose for all enantiomers and CSP were computed showing hydrophobic interactions, H-arene and π-π stacking to be those most involved for the binding of the analytes. H-bonding occurs between the OH at the chiral center and the carbonyl in CSP. Overall, the list of major analyte-CSP interactions includes the following:

- H-bonding between the -OH at the chiral center and the carbonyl in CSP
- H-bonding between carbazole-N and the carbonyl group in CSP
- H-bonding between -OH in the substituted phenyl with carbonyl in CSP
- H-π interactions between the analyte’s (substituted) phenyl moiety and the six membered ring in CSP
- H-π bonds between phenyl and carbazole rings and six membered ring in CSP
- π-π interaction between analyte phenyl and the carbonyl group in CSP
- π-π interaction between analyte phenyl and phenyl in CSP

The scores of poses for the respective enantiomers were −25.11 vs. −22.71 for CAR, −22.71, vs. −23.78 for 4′OHC, −22.08 vs. −24.45 for 5′OHC, −18.07 vs. −20.12 for DMC, and −15.01 vs. −15.12 for MET enantiomers. Differences in the scores of enantiomers of the same compound represent different binding affinities to derivatized cellulose explaining enantiomer separation on the CSP. It was also observed that the critical peaks (4′OHC enantiomers) were the ones with the lowest difference in their score (−1.07) compared to CAR and its other metabolites. MET used as the IS eluted first with its enantiomers unresolved, as proven by the docking study, where the enantiomers have very similar scores at this particular CSP and the least negative ones. In Table 4, significant physicochemical parameters, the derived CSP-binding scores, score differences for enantiomer pairs as well as the optimized chromatographic parameters are given for all analytes.

### 2.5. Experimental Study to Estimate the Enantiomer-Elution Order-No Prediction Possible from Docking Study

**Figure 10:**
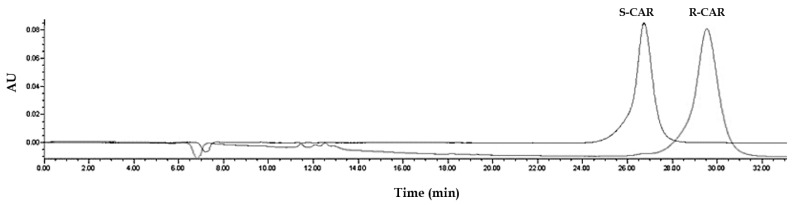
Experimental determination of the elution order: separate runs using the same chromatographic conditions for *S*-CAR (retention time, 26.7 min) and *R*-CAR (retention time, 30.5 min). The amount injected per run: 1 ng = 10 µL of a 100 ng/mL solution. The chromatographic conditions are: mobile phase consisting of 80% organic modifier (87% acetonitrile: 13% methanol) and phosphate buffer pH 7, delivered at a flow rate of 0.5 mL/min, temperature at 25 °C.

Out of the parameters obtained in the docking study, no prediction of the elution order is possible: *R*-CAR should elute earlier than *S*-CAR due to inferior binding to the CSP represented in a higher binding score for the S enantiomer (−25.11 for *S* vs. −20.60 for *R*). It was proven experimentally by running CAR enantiomers separately (Figure 10) that *S*-CAR elutes before *R*-CAR.

Overall, although there was a tendency for scores to be higher for more lipophilic analytes, elution order of the different *rac*-analytes was not explained by the scores obtained in the docking study. This might be due to the fact that solvent effects (mobile phase composition) were not taken into consideration in the current docking study. 

It is assumed that the elution order is not determined by the mobile phase composition, but rather determined by the type of the chiral stationary phase, and that in a set of structurally closely related compounds elution order is similar for all species. In the case of CAR, studies with isolated metabolite *S*- and *R*-enantiomers on amylose-based CSPs indicated that the enantiomer elution order is similar for the parent compound and its metabolites. It is assumed that this is also the case for cellulose-based CSPs. Hence, it was concluded that the elution order is always *S* before *R*.

### 2.6. Plasma Concentrations in Patients

Although extracts from patients’ samples produced more baseline “noise” in the chromatograms, it was possible to apply the optimized method to biological material. In the analyzed samples obtained after a 25 mg dose of CAR racemate, *R*-CAR and *S*-CAR plasma concentrations were largely similar, i.e., the *S/R* ratios were close to 1. The sum of the two enantiomers does not exceed 100 ng/mL in the patients investigated (Table 3).

## 3. Discussion and Conclusions

### 3.1. Method Development and Validation

Using cellulose-based CSP, the enantiomer resolution of each of the four single *rac*-analytes was possible, i.e., it was possible to achieve an enantiomer resolution with each of the enantiomeric pairs when injected as single pure reference. The most lipophilic CAR enantiomers were usually easily separated from their metabolites. However, separation in between the group of three oxidative metabolites, particularly the two methoxyphenyl-hydroxylated metabolites, was more difficult, since there was considerable partial up to complete overlap between the respective twin peaks within the group of these compounds. 

In the current studies, the utilized systematic DoE approach proved to be applicable and resulted in a time-efficient optimized method of analyte separation and chiral resolution. Within the response surface, the main effects of the most significant factors—percentage of organic modifier, buffer pH, and organic modifier composition—were investigated and a response surface was created using central composite design.

The novel, simple, robust, and sensitive UHPLC-UV method that resulted for the simultaneous enantioselective quantification of CAR along with its active oxidative metabolites (4′OHC, 5′OHC, and DMC) represents a highly reliable method in a stable chromatographic system. It was proven suitable for an application to biomaterials, e.g., for human plasma, in the current study. However, the temperature needs to be controlled, and the CSP employed needs to be handled carefully and be regenerated regularly, particularly when, after liquid–liquid extraction, plasma extracts as opposed to “clean” standard solutions are injected onto them. Reconstituted extracts, depending on the extraction solvent composition, contain co-extracted lipophilic material, which may affect column performance on the longer run. By implementing regular regeneration cycles it was/is possible to maintain the quality of the stationary phase and the chromatographic outcome throughout an extended study period.

The UV-detection mode was well applicable, however, the method may be further developed and possibly LOD and LOQ be optimized by applying, e.g., fluorometric measurement of the eluates. In other CAR studies with reversed-phase mode HPLC, for instance, 280 nm/345 nm (λ_max_ ex/λ_max_ em), light source: xenon lamp) was selected, in order to monitor the intrinsic fluorescence of the parent CAR and the metabolites in the eluate [13,14,16]. Application of fluorescence monitoring may improve specificity and LOD and provide some advantage towards the current absorbance monitoring of the eluates at 240 nm. This may be useful for studies with administration of lower doses of *rac*-CAR.

Overall, the chromatographic system provides sufficient enantiomer resolution at an acceptable total runtime. Additional advantages of this method include small sample volume (0.5 mL), simple plasma extraction procedure with high extraction recovery, and a readily available IS.

Therefore, the method may be very useful and a suitable alternative bioanalytical assay when performing enantiomer-kinetic studies on the adrenoceptor–antagonist CAR and its metabolites under clinical settings.

### 3.2. Estimation of the Elution Order of Eutomer vs. Distomer and the Respective Metabolite Enantiomers

It was not possible to determine the elution order of CAR enantiomers in silico. It was, however, assumed that the elution order is similar for metabolites and the parent CAR, since in preliminary studies performed with traces of the single enantiomers of the three oxidative metabolites under slightly different conditions (i.e., on HPLC, not UHPLC, and with amylose-based CSP) the capacity factors of the *S*-enantiomers were always below those of the respective *R*-enantiomers. It may be concluded that for the cellulose-based CSP as well and for all analytes the *S*-enantiomer always elutes prior to that of the respective optical antipode (as is the case for CAR enantiomers on Chiralpak IB N-5). Moreover, studies in patients, for which a higher metabolic clearance for *S*-CAR than for *R*-CAR with preferential formation of the *S*-enantiomers of the metabolites was expected, yielded higher levels for all first-eluting metabolite enantiomers. Alternatively, *experimental approaches* are feasible, in order to confirm that the metabolite enantiomers with smaller capacity factors are representing the respective *S*-enantiomers: (1) in-vitro metabolism or (2) animal metabolic studies with either *R*-CAR *or S*-CAR as substrates. This is a possible approach, since no metabolic stereoinversion occurs for such drug substances and only metabolites of the respective enantiomer are formed.

With respect to the application of the method, the individual random samples taken from different multimorbid Egyptian patients yield CAR *S/R* ratios somehow different from those obtained in our previous studies on CAR in healthy Caucasian volunteers [4]. It is well known that for high-extraction ratio drugs variabilities may be high with respect to the plasma concentration–time profiles. Generally, the higher total pre-systemic extraction, the higher is the *S*-selectivity in clearance. Genetic variations are known to correlate with clearance differences (the clearance of any subject can be predicted as the sum of a CYP2D6-independent clearance and two CYP2D6 allele-specific clearances [9]) and may be considered when interpreting kinetic data.

In general, when *rac*-CAR is administered, any reduction in first-pass effect of the high-extraction ratio drug CAR (because of decreased hepatic clearance, i.e., due to interfering comedication or in liver-impaired subjects) leads to *S/R* ratios closer to 1, which means decreased stereoselectivity. Overall, the concentration data were well within the ranges found in other studies and the assay proved useful.

Physicochemical parameters of molecules and in-silico studies are—in general and in many specific respects (receptor affinities, membrane passage/drug targeting, drug metabolism, assay development)—enormously helpful and provide in-silico predictions. In-silico studies presented here included docking strategies, yet in vacuo, i.e., without taking into consideration that the mobile phase (e.g., water molecules) may have an impact as well. Therefore, more elaborate models should include solvent aspects, particularly for aqueous reversed-phase systems. This might also help to better elucidate factors, which determine the elution order in silico, which was not possible here, although enantiomer resolution was significantly associated with the calculated absolute value of the score difference. This issue will need further and more detailed investigation.

Nevertheless, the docking simulation, which was performed for all analytes on tris (3,5-dimethylphenyl carbamate)-derivatized cellulose, provided some insight on the mechanisms of interaction and of analyte separation and chiral recognition: It was possible to identify major binding areas on the CSP.

The retention behavior of the analytes (adjusted retention times and capacity factors) does correlate with lipophilicity parameters (clogP), and enantiomer resolution is better with higher retention (Table 5). However, the elution order (Figure 7) does not correlate with any of the investigated physicochemical or hybrid parameters and cannot be predicted specifically. This implies that the initial selection of appropriate (ideally also structurally related) internal standard candidates may be based on this and few other easily accessible physicochemical molecular parameters.

## 4. Materials and Methods

### 4.1. Chemicals

CAR racemate and enantiomers together with its major metabolites (4′OHC, 5′OHC, DMC) were available in our labs. CAR was of pharmacopoeial purity (Ph. Eur. 9th Ed.). Its metabolites and its enantiomers had been obtained from the inventor/manufacturer (Boehringer Mannheim/Hoffmann-LaRoche, Mannheim, Baden-Wurttemberg, Germany), been stored in portions at low temperatures (<−20 °C) and continuously been protected from light and humidity. Absence of degradation products was confirmed via chromatography.

Methanol, acetonitrile (both HPLC grade), ammonium acetate, dipotassium phosphate and potassium dihydrogen phosphate were purchased from Sigma-Aldrich (Darmstadt, Germany). Ultra-pure water was always freshly prepared throughout the analyses using Purelab UHQ water (ELGA, Woodridge, IL, USA).

### 4.2. Equipment and Software

The chromatographic system consisted of a Waters Acquity UHPLC-PDA System (Waters Corporation, Milford, MA, USA) coupled to a photodiode array (PDA) detector set at 240 nm (λ_max_ of CAR), separations were performed on a Chiralpak IB N-5 column (250 × 4.6 mm, 5 µM) from DAICEL corporation, Illkirch Cedex, France. The optimized mobile phase used was as follows: organic modifier set at 80% (with acetonitrile 87% and methanol 13%) and 20% potassium phosphate buffer pH of 7, flow rate of 0.5 mL/min and temperature at 25 °C. Data acquisition was performed using Waters Empower 2 chromatography data software. Integration was performed using “ApexTrack” integration, where curvature is measured by calculating the 2nd derivative of the chromatogram.

Software for statistical analyses and docking studies: The software used were Minitab17 Statistical Software (Coventry CV3 2TE, Binley Woods, UK) for multivariate analysis and response surface methodology and the MOE software (Molecular Operating Environment, Version 2019, from Chemical Computing Group, Montreal, QC, Canada) for the docking studies and the estimation of molecular physicochemical parameters, with which chromatographic behavior might correlate.

### 4.3. Stock Solutions, Dilutions for Method Optimization, and Plasma Standard and QC Sample Preparation

Concentrated and diluted stock solutions were prepared in methanol (e.g., *rac*-CAR (1000 ng/mL), internal standard (IS) *rac*-metoprolol (MET) (100 ng/mL)). Then, 0.5-mL plasma aliquots were spiked yielding 5 standard samples having concentrations of 5.0, 12.5, 25.0, 50.0, 100.0 ng/mL of each CAR enantiomer.

Concentrated and diluted stock solutions of CAR metabolites (*rac*-4′OHC, *rac*-5′OHC and *rac*-DMC) were also prepared in methanol. Then, 0.5 mL plasma aliquots were spiked yielding plasma standard samples having concentrations of 0.0, 1.25, 2.50, 5.00, 10.00, 20.00 ng/mL metabolite enantiomer.

The *rac*-CAR quality-control (QC) samples were prepared from blank human plasma yielding low-, middle-, and high-concentration samples (QC; 7.0, 30.0 and 95.0 ng/mL *rac*-CAR, respectively, i.e., 3.5, 15.0, 47.5 ng/mL for each enantiomer), followed by storage at −80 °C until analysis for estimation of intra- and inter-day precision and accuracy. Similarly, for *rac*-metabolites (4′OHC, 5′OHC and DMC) low, middle-, and high-QC samples were prepared (with concentrations of 2.0, 7.0 and 18.0 ng/mL, respectively, i.e., 1.0, 3.5, 9.0 ng/mL for each enantiomer).

For studies designed to estimate the elution order of the enantiomers, *R*- and *S*-CAR reference solutions were also prepared in methanol (100 ng/mL).

### 4.4. UHPLC-UV Method Development Using a DoE Approach

#### 4.4.1. Screening Design

**Figure 11:**
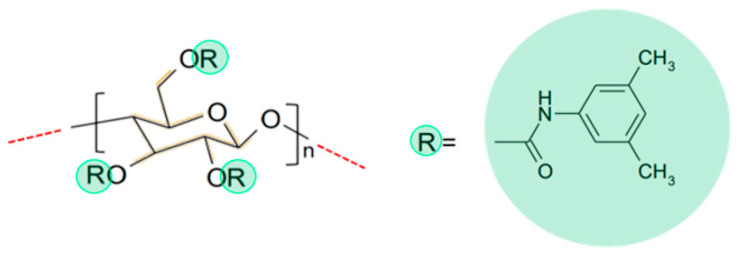
Chemical structure of the chiral stationary phase tris (3,5-dimethylphenyl carbamate)-derivatized cellulose immobilized on 5 µM silica-gel in the Chiralpak IB N-5 column used for separation and chiral resolution of analytes in the current study. Cellulose consists of β(1→4) linked d-glucose units.

Enantiomer separation of CAR in presence of its metabolites DMC, 4′OHC, 5′OHC was investigated directly using UHPLC on a cellulose based CSP Chiralpak IB N-5 column stationary phase (Figure 11) in reversed-phase mode by a DoE approach. The DoE approach was implemented to explore the effect of changing several chromatographic parameters on two responses; separation (resolution) and retention time [24,25].

Initial screening of various chromatographic parameters was performed using fractional factorial design. The five potential factors for screening included 2 categorical factors (organic modifier composition and buffer type) and 3 numerical factors (organic modifier %, buffer pH and flow rate) (Table 5). Usually, temperature is also involved as factor to be investigated, however, according to the manual of the Chiralpak columns, temperature should be kept between 5 and 25 °C when these columns are used at pH > 7. For the five factors, two levels (high and low) were chosen. Organic modifier composition, organic modifier %, buffer type, buffer pH levels were chosen according to the recommended typical start-up reversed-phase conditions for Chiralpak columns with basic analytes. Flow rate was also investigated as a general factor known to significantly affect the retention time as well as the resolution. The racemic analytes (50 ng/mL in methanol) were injected throughout the screening design runs with an injection volume of 10 µL.

**Table 5:** Lower and higher levels of the *Initial screening* design for simultaneous separation and enantiomer resolution of CAR and its metabolites.

Factor	Low Level (−1)	High Level (+1)
Organic modifier composition	Acetonitrile	Acetonitrile: Methanol (1:1, *v*/*v*)
% of Organic modifier	60%	80%
Buffer type	20 mM Ammonium acetate buffer	20 mM potassium Phosphate buffer
Buffer pH	7.0	8.0
Flow rate	0.5	0.7

#### 4.4.2. Central Composite Optimization Design

After determining the most influential parameters on the enantiomer resolution and retention times for all analytes, central composite design (CCD) with 3 finally selected factors (X_1_, X_2_, X_3_) was employed to define the response surface (Table 6). The CCD is a 2-level full factorial design having a total of 20 runs (Table 1) involving 8 cube points, 6 center points and 6 axial points. Similar amounts of analytes were injected throughout the optimization study (racemate concentration of methanolic solutions 50 ng/mL, injection volume 10 µL).

#### 4.4.3. Regression Analysis

ANOVA was carried out first for resolution (R_s_) and adjusted retention time (t’_R_) as the responses to judge significance of the 3 factors (X_1_, X_2_ and X_3_) along with their quadratics and their 2-way interactions. Sequential removal of insignificant terms in the models was carried out (*p* > 0.05) until no further increase in the R^2^ was observed. The model was evaluated according to its SE, R^2^, R^2^_adj_, and R^2^_pred_. Main effects plot and contour plots were created for both response models. Normal probability plots of residuals, residuals-vs.-fit plots, histogram of residual and residuals-vs.-order of data were also generated to examine the goodness-of-fit in regression models.

#### 4.4.4. Validation of Predictability of Models

To evaluate the accuracy and prediction ability of the models, five experimental runs were conducted with factors lying in the robust surface of highest resolution and lowest retention time by the overlapping contour plots from the CCD. Experimental values were then compared to predicted ones.

#### 4.4.5. Global Solution for Optimum Run Conditions

After testing the model predictability, a global optimum for chromatographic parameters (X_1_, X_2_, X_3_) was computed to offer highest resolution and lowest retention time based on the final regression models. The responses at predicted optimum conditions values were tested experimentally and compared to the predicted ones.

### 4.5. Biological Material: Separation of Plasma from Blood

Fresh pooled blank blood was purchased from Vacsera Co., Cairo, Egypt and transferred into commercially available EDTA-treated containers. Blood samples obtained from patients were also collected in EDTA-containing tubes. Blood was kept cool and then centrifuged at 2000 g for 15 min. Following plasma separation, it was immediately transferred into clean polypropylene tubes. The samples were always kept at 2–8 °C while handling. Aliquots (0.5 mL) of plasma were either used immediately to prepare plasma standards and/or kept frozen at −20 °C until used.

### 4.6. Sparse Blood Sampling from Patients to Test Assay Applicability

In order to prove the applicability of the method under clinical settings, samples were obtained at random from 6 multimorbid cardiovascular patients (3 male, 3 female; dose: 25 mg *rac*-CAR), who were of middle to high age and for whom comedication was not excluded (Table 7). After collection, plasma was obtained and the samples stored frozen until analysis.

### 4.7. Plasma Sample Work-Up: Liquid-Liquid Extraction of CAR and Its Major Oxidative Metabolites from Patient Plasma

Sodium hydroxide solution (1.0 M; 0.5 mL) was added to a 0.5 mL-aliquot of blank plasma, of standard plasma sample, of QC sample, or of a patient’s plasma sample, respectively. After vortex mixing for 5 s, 50 µL of the IS stock (100 ng/mL) and 300 µL methanol were added (again vortexed) followed by 6 mL of a mixture of diethyl ether and ethyl acetate (3:1, *v*/*v*) for liquid-liquid extraction. The mixture was vortexed for 30 s and centrifuged at 3000× *g* for 3 min. The organic layer was transferred into another tube and evaporated to dryness, at room temperature under a stream of nitrogen. The residue was reconstituted in 100 μL of methanol, and a 10-μL aliquot was injected into the UHPLC-UV system [26].

### 4.8. Preliminary Method Validation for CAR Enantiomers and the Enantiomers of Its Oxidative Metabolites in Plasma

The response surfaces obtained for all enantiomers confirm optimum resolution and method robustness. Therefore, the optimized chromatographic system was basis for studies on biological materials.

According to the ICH M10 guidelines for bioanalytical methods [23], the developed method was validated to assure the reliability of the results of the analysis for different parameters, including system suitability, linearity, limit of detection (LOD), limit of quantification (LOQ), accuracy, precision and robustness. Linearity was assessed/confirmed by plotting enantiomer calibration curves of CAR for the range 5.0 to 100.0 ng/mL, 4′OHC, 5′OHC, and DMC for the range 1.25 to 20.00 ng/mL. The percentage relative standard deviations (RSD %) of the regression coefficient were calculated. LOD and LOQ were estimated using the following formulas: LOD = 3.3 σ/S and LOQ = 10 σ/S, where σ is the standard deviation of the response and S is the slope of the calibration curve. Accuracy was ascertained by the recovery method using QC samples. Recovery %, RSD %, and standard error (SE) were calculated. Precision was determined by measuring repeatability, intra-day as well as inter-day precision of peak areas of the enantiomers for parent compound and the metabolites. The method robustness was assessed by visualization of robust areas in the response surface (Figure 3 and Figure 4) for factors optimized in the CCD (organic modifier %, organic modifier composition and buffer pH).

Extraction recoveries of CAR and its metabolites from spiked human plasma samples were compared with analytical standards of the same concentration. The average relative extraction yields were calculated for the three QC samples for each analyte: For each concentration level, the extracted and unextracted samples were analyzed in triplicate and the arithmetical mean was calculated.

### 4.9. Computational Study of the Binding of Analytes to the Chiral Selector

**Figure 12:**
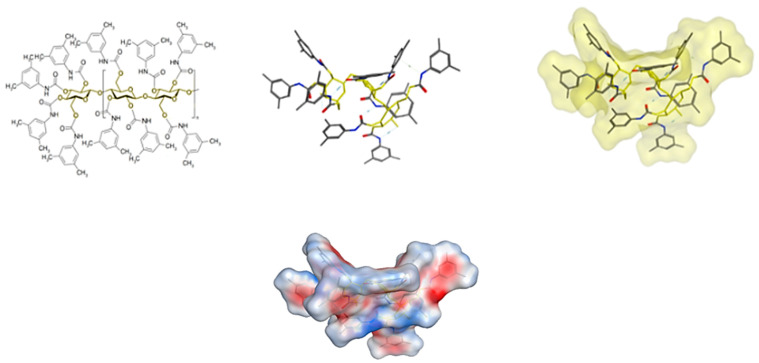
Molecular structure and 3D structures of tris (3,5-dimethylphenylcarbamate)-derivatized cellulose, which was used as chiral selector and, hence, as binding partner for the analyte-CSP docking experiments. Upper part: **Left**: 2D image of a terminal 3-glucose-unit segment of derivatized cellulose (consisting of β(1→4) linked d-glucose units), where the terminal unit has 4 carbamate residues. The derivatized cellobiose subunit is repeated forming the derivatized cellulose polysaccharide. The cellulose core of the molecule (3-unit segment) is marked yellow. **Middle**: A middle 3-glucose-unit segment as 3D stick image. Cellulose units are marked yellow. **Right**: 3D stick image similar to that in the middle, yet with yellow transparent surface and-Lower part-with *colored* surface (TPSA), where red represents more negative and blue positive areas.

In order to provide more insight into the mechanism of the enantiomeric separation of CAR and its metabolites on CSP of Chiralpak IB N-5 column, a docking study was performed using MOE 2019. The CSP-analyte complex was visualized using global docking for virtual modeling of the chiral recognition process. Initial conformational search was performed for CAR, 4′OHC, 5′OHC, DMC and MET enantiomers using rejection limit of 100, iteration limit of 100 and an RMS Gradient of 0.005. Conformational search yielded a total of 10 low energy conformers for each enantiomer that was included into docking studies on the CSP. The analytes’ enantiomer conformers were docked separately on energy-minimized cellulose tris (3,5-dimethylphenyl carbamate) (Figure 12) using Global docking. The force field, “Amber 10”, was used throughout the docking experiment. “Triangle matcher” was used as a placement method and “induced fit” was set to be the refinement method creating a database of the best poses for each enantiomer. The scoring function used was London dG and includes clogP, TPSA (topological polar surface area), and VdW*area* (Van der Waal surface area) as major parameters. The poses with the best (= most negative) score were visualized bound to the CSP for a better understanding of the different binding modes and interactions for both enantiomers.

## Figures and Tables

**Table 1:** Run conditions (20) for the central composite optimization design and the resolution (R_s_) and retention time of the late eluting peak (t’_R_) as responses.

Run Order	% Organic Modifier	Buffer pH	% Acetonitrile in Organic Modifier	R_s_	t’_R_
1	80	7.0	80	0.77	17.6
2	70	7.5	70	0.60	45.1
3	80	8.0	80	0.77	23.1
4	70	7.5	70	0.63	47.1
5	70	8.2	70	0.64	68.0
6	70	7.5	87	0.70	35.6
7	70	6.7	70	0.46	58.8
8	53	7.5	70	0.45	175.8
9	60	8.0	60	0.74	176.4
10	70	7.5	70	0.60	46.3
11	60	8.0	80	0.67	92.4
12	60	7.0	80	0.00	81.6
13	70	7.5	70	0.64	47.3
14	70	7.5	70	0.65	47.8
15	87	7.5	70	0.56	15.4
16	70	7.5	53	0.66	71.9
17	80	8.0	60	0.52	26.5
18	60	7.0	60	0.00	151.8
19	70	7.5	70	0.60	45.6
20	80	7.0	60	0.46	24.40

**Table 2:** Selected assay validation parameters.

Parameters	CAR	4′OH-C	5′OH-C	DMC
Linearity range (ng/mL)	5–100	1.25–20	1.25–20	1.25–20
Regression equation X: Concentration (ng/mL) Y: Peak area ratio	Y = 0.0508x − 0.0029	Y = 0.1374x − 0.0397	Y = 0.1500x − 0.0329	Y = 0.0935x + 0.0705
SD of slope	1.320 × 10^−4^	7.000 × 10^−4^	2.050 × 10^−3^	2.020 × 10^−3^
SD of y-intercept	6.900 × 10^−5^	3.120 × 10^−3^	2.630 × 10^−3^	2.290 × 10^−3^
*R^2^*	0.996	0.997	0.997	0.994
SD of *R^2^*	2.030 × 10^−5^	9.180 × 10^−4^	9.150 × 10^−4^	5.460 × 10^−4^
LOD	0.004	0.068	0.053	0.074
LOQ	0.014	0.227	0.175	0.245

Linearity of calibration curves for CAR, 4′OHC, 5′OHC, and DMC (n = 9) and calculated LOD and LOQ for all analytes (given as average value of the two enantiomers for a better overview; *R/S*-ratios were always close to 1 or equal to 1 (range, 0.98–1.03) and the characteristics very similar for the two individual enantiomers).

**Table 3:** Plasma concentrations of CAR and metabolite enantiomers in randomly collected blood samples obtained from cardiovascular patients after oral administration of 25 mg racemic CAR (12.5 mg = 30.75 µmol per enantiomer) at the respective times of blood sampling.

Patient Number	Sampling Time (hr)	Plasma Concentration (ng/mL)															
		CAR				DMC				4′OHC				5′OHC			
		*S*-(−)	*R*-(+)	*S/R*	*rac*-	*S*-(−)	*R*-(+)	*S/R*	*rac*-	*S*-(−)	*R*-(+)	*S/R*	*rac*-	*S*-(−)	*R*-(+)	*S/R*	*rac*-
1	0.5	20.28	20.70	0.98	40.98	-	-	-	-	-	-	-	-	-	-	-	-
2	1.0	50.01	48.39	1.00	98.40	3.27	2.40	1.36	5.67	-	-	-	-	-	-	-	-
3	2.0	-	-	-	-	9.92	6.06	1.51	15.98	13.74	4.51	3.05	18.25	11.48	8.20	1.40	19.68
4	4.0	-	-	-	-	8.52	4.93	1.97	13.45	16.06	4.98	3.22	21.04	14.06	9.47	1.49	23.53
5	9.0	-	-	-	-	5.02	2.82	2.41	7.84	8.18	3.54	2.32	11.72	8.09	6.09	1.33	14.18
6	12.0	-	-	-	-	2.73	1.52	1.97	4.25	5.29	1.96	2.70	7.25	5.28	3.78	1.37	9.06

**Table 4:** Molecular/physicochemical parameters of analytes (CAR enantiomers, metabolite enantiomers, and internal standard enantiomers), individual CSP-binding scores, score differences of analyte pairs, and chromatographic characteristics under optimized conditions.

Molecule	m.w. (g/mol)	cLogP	vdWSA	TPSA	Individual Binding Score (Sc)	*R*-vs.-*S* Score-Difference	Capacity Factor k	Separation Factor (α)	Resolution (R_s_)
*R*-CAR	406.5	3.90	668	75.74	−20.60	4.51	4.28	1.12	best resolution 2.4
*S*-CAR	406.5	3.90	661	75.74	−25.11		3.72		
*R*-5′OHC	422.5	3.40	684	95.97	−22.08	2.37	1.41	1.04	1.2
*S*-5′OHC	422.5	3.40	683	95.97	−24.45		1.31		
*R*-DMC	392.5	3.49	640	86.74	−18.07	2.05	2.35	1.04	1.0
*S*-DMC	392.5	3.49	640	86.74	−20.12		2.21		
*R*-4′OHC	422.5	3.40	684	95.97	−22.71	1.07	1.22	1.07	worst resolution (critical peaks) 0.8
*S*-4′OHC	422.5	3.40	687	95.97	−23.78		1.08		
*R-MET (IS.)*	267.4	1.99	525	50.72	−15.01	0.11	0.55	1.0	no resolution0
*S-MET (IS)*	267.4	1.99	523	50.72	−15.12		0.55		

In fitted regression models (relative weights always 1) including capacity-, separation- and resolution factors well as the docking score, the following relative importance of the following major descriptors were found: for capacity factor, PSA 0.4 and vdWSA 0.54; for separation factor, TPSA 0.39 and vdWSA 0.56; for resolution factor, TPSA 0.80; for docking score, vdWSA 0.85. [vdWSA, Van der Waals surface area as area available for electrophobic interactions in the conformation bound to the CSP; TPSA, topological polar surface area; individual binding score, Sc; IS, internal standard].

**Table 6:** Lower and higher levels of the optimization design for simultaneous separation of CAR and its metabolites.

Factor	Low Level	High Level
X_1_: % of organic modifier	60	80
X_2_: Buffer pH	7.0	8.0
X_3_: Organic modifier composition: % Acetonitrile of the total organic modifier (remaining is methanol)	60%	80%

**Table 7:** Patients’ data (patient number, gender, age, weight and diagnosis).

Patient Initials	Number	Gender	Age (yrs)	Weight (kg)	Diagnosis
HH	1	F	51	87	Ischemic heart failure Diabetes
MG	2	F	66	91	Hypertension Gastritis Anemia
RS	3	M	55	89	Myocardial infarction Diabetes
EI	4	M	47	76	Hypertension Gastritis
AS	5	F	53	95	Hypertension Gout
KS	6	F	62	85	Hypertension Diabetes

## Data Availability

Not applicable.

## Sample Availability

Samples of all the prepared compounds are available from the authors.
